# Obsessive-compulsive disorder and suicide: a longitudinal study in Taiwan

**DOI:** 10.1017/S2045796024000477

**Published:** 2024-09-23

**Authors:** Mu-Hong Chen, Tai-Long Pan, Chih-Ming Cheng, Wen-Han Chang, Ya-Mei Bai, Tung-Ping Su, Tzeng-Ji Chen, Shih-Jen Tsai

**Affiliations:** 1 Department of Psychiatry, Taipei Veterans General Hospital, Taipei, Taiwan; 2 Department of Psychiatry, College of Medicine, National Yang Ming Chiao Tung University, Taipei, Taiwan; 3 School of Traditional Chinese Medicine, Chang Gung University, Taoyuan, Taiwan; 4 Research Center for Chinese Herbal Medicine and Research Center for Food and Cosmetic Safety, College of Human Ecology, Chang Gung University of Science and Technology, Taoyuan, Taiwan; 5 Liver Research Center, Chang Gung Memorial Hospital, Taoyuan, Taiwan; 6 Department of Psychiatry, General Cheng Hsin Hospital, Taipei, Taiwan; 7 Department of Family Medicine, Taipei Veterans General Hospital, Taipei, Taiwan; 8 Institute of Hospital and Health Care Administration, National Yang Ming Chiao Tung University, Taipei, Taiwan; 9 Department of Family Medicine, Taipei Veterans General Hospital, Hsinchu Branch, Hsinchu, Taiwan

**Keywords:** major psychiatric comorbidities, obsessive-compulsive disorder, suicide, Taiwan

## Abstract

**Aims:**

Research evidence has established an association of obsessive-compulsive disorder (OCD) with suicidal thoughts and suicide attempts. However, further investigation is required to determine whether individuals with OCD have higher risk of death by suicide compared with those without OCD.

**Methods:**

Of the entire Taiwanese population, between 2003 and 2017, 56,977 individuals with OCD were identified; they were then matched at a 1:4 ratio with 227,908 non-OCD individuals on the basis of their birth year and sex. Suicide mortality was assessed between 2003 and 2017 for both groups. Time-dependent Cox regression models were used to investigate the difference in suicide risk between individuals with versus without OCD.

**Results:**

After adjustment for major psychiatric comorbidities (i.e., schizophrenia, bipolar disorder and major depressive disorder), the OCD group had higher risk of suicide (hazard ratio: 1.97, 95% confidence interval: 1.57–2.48) during the follow-up compared with the comparison group. Furthermore, OCD severity, as indicated by psychiatric hospitalizations due to OCD, was positively correlated with suicide risk.

**Conclusions:**

Regardless of the existence of major psychiatric comorbidities, OCD was found to be an independent risk factor for death by suicide. A suicide prevention program specific to individuals with OCD may be developed in clinical practice in the future.

Obsessive-compulsive disorder (OCD) is a debilitating and chronic neuropsychiatric disorder, and its lifetime prevalence of 1%–3% (Fontenelle and Yucel, 2019; Hirschtritt *et al.*, 2017). This disorder is characterized by intrusive, distressing thoughts known as obsessions and time-consuming, repetitive ritualistic behaviours referred to as compulsions, all of which greatly impair daily functioning (Fontenelle and Yucel, 2019; Hirschtritt *et al.*, 2017). According to the World Health Organization, OCD is ranked as the 4th most debilitating disorder worldwide in the 15–49 years age group, 10th in the 50–69 years age group and 21st in the 70 years age group (Baxter *et al.*, 2014; Murray and Lopez, 1996). However, OCD is often underdiagnosed in primary care settings and is frequently undertreated (Fontenelle and Yucel, 2019; Hirschtritt *et al.*, 2017).

An increasing body of evidence is indicating that individuals with OCD are more prone to experiencing suicidal symptoms – including suicidal thoughts, suicide attempts and suicide deaths – compared with those without OCD (Fernandez de la Cruz *et al.*, 2017; Kamath *et al.*, 2007; Ouazzani Housni Touhami *et al.*, 2023; Samuels *et al.*, 2023). Samuels *et al.* (2023) analysed the lifetime prevalence of suicide attempts in 515 adults with OCD who also had a history of major depressive disorder. They found that 64 (12%) of the participants had a lifetime history of suicide attempts. They further suggested that obsessions involving violent or horrific images were particularly associated with suicide attempt risk (Samuels *et al.*, 2023). A cohort study of 100 inpatients with OCD aged 27.03 ± 9.92 years demonstrated that the lifetime and current rates of suicidal thoughts were 59% and 28%, respectively, and that the lifetime rate of suicide attempts was 27% (Kamath *et al.*, 2007). Kamath *et al.* (2007) demonstrated that OCD-related depression and feelings of hopelessness were significantly correlated with suicidal behaviour. Ouazzani Housni Touhami *et al.* (2023) indicated that patients with schizophrenia and comorbid OCD, commonly classified as schizo-obsessive, exhibited more severe psychotic symptoms, had higher levels of anxiety and depression and made more suicide attempts compared with those without comorbid OCD. A population-based longitudinal study of 36,788 Swedish patients with OCD reported that 545 of these patients died by suicide and 4,297 had attempted suicide, with odds ratios (ORs) of 9.83 and 5.45, respectively, when the reference group comprised individuals without OCD (Fernandez de la Cruz *et al.*, 2017). A nationwide cohort study of 61,378 people with OCD and 613,780 non-OCD people revealed that people with OCD had an increased risk of suicide (hazard ratio [HR]: 4.90, 95% confidence interval [CI]: 4.40–5.46) compared with non-OCD people (Fernandez de la Cruz *et al.*, 2024). Fernandez de la Cruz *et al.* (2024) further indicated a consistent finding of an increased likelihood of suicide in patients with OCD across different subgroups of psychiatric comorbidities, including schizophrenia, bipolar disorder and major depressive disorder. However, the aforementioned studies have primarily focused on Western populations, which may limit the generalizability of their findings to Asian populations. Additionally, further investigation is required to determine whether OCD represents an independent risk factor for suicide irrespective of the existence of major psychiatric comorbidities such as schizophrenia and major depressive disorder.

In the present study, we employed the Taiwan National Health Insurance Research Database (NHIRD), which encompasses data on the entire population of Taiwan, and adopted a longitudinal study design to examine the independent role of OCD in suicide risk after adjustment for major psychiatric comorbidities. We hypothesized that individuals with OCD were at elevated risk of dying by suicide during the follow-up compared with those without OCD, independent of the existence of schizophrenia, major affective disorders and neurodevelopmental disorders.

## Methods

### Data source

The Taiwan Health and Welfare Data Science Center of Ministry of Health and Welfare audits and releases the NHIRD, which consists of comprehensive healthcare data of >99.7% of the entire Taiwan population, for research purposes. Individual medical records included in the NHIRD are anonymous to protect patient privacy. In the current study, we linked the Longitudinal Health Insurance Database of the NHIRD, which includes all medical records between 2003 and 2017 of the entire Taiwanese population (*n* = 29,253,529), and the Database of All-cause Mortality, which includes all-cause mortality records between 2003 and 2017 of the entire Taiwanese population, for the analyses of suicide risk among Individuals with OCD. The *International Classification of Diseases, 9th or 10th Revision, Clinical Modification* (*ICD-9-CM* [2003–2014] or *ICD-10-CM* [2015–2017]) are used in the clinical practice in Taiwan. The institutional review board of Taipei Veterans General Hospital approved the study protocol and waived the requirement for informed consent because deidentified data were used in this study and no participants were actively enrolled. The NHIRD has been used in numerous epidemiological studies in Taiwan (Chen *et al.*, 2013; Cheng *et al.*, 2018; Hsu *et al.*, 2023; Zhang *et al.*, 2021).

### Inclusion criteria for individuals with OCD and the comparison group

**Figure 1.**
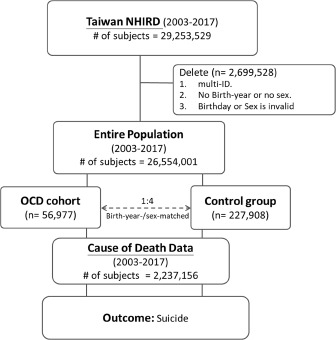
Study flowchart.

NHIRD: Taiwan National Health Insurance Research Database; OCD: obsessive compulsive disorder.

Individuals who had a diagnosis of OCD (ICD-9-CM code: 300.3 or ICD-10-CM code: F42) given by board-certified psychiatrists at least twice were included as the OCD group (Fig. 1). To reduce the confounding effects of age and sex, a 1:4 OCD–non-OCD matched analysis was conducted based on birth year and sex. The comparison group was randomly identified from the entire Taiwanese population after those who had an OCD diagnosis at any time in the database were excluded (Fig. 1). The urbanization level of residence (levels 1–4, most to least urbanized) was assessed as a proxy for healthcare availability in Taiwan (Liu *et al.*, 2006). The OCD diagnosis was regarded as a time-dependent variable. For instance, we coded the OCD status as 0 before 2005 and as 1 since 2005 if the OCD diagnosis occurred in 2005 during the study period between 2003 and 2017. Suicide was identified between 2003 and 2017 from the Database of All-cause Mortality. For individuals with OCD and matched individuals, Charlson Comorbidity Index (CCI) scores were calculated. The CCI comprising 22 physical conditions was also assessed to determine the systemic health conditions of all enrolled subjects (Charlson *et al.*, 1987). Furthermore, because OCD may be comorbid with schizophrenia (ICD-9-CM code: 295 or ICD-10-CM code: F20, F25), bipolar disorder (ICD-9-CM codes: 296 except 296.2, 296.3, 296.9 and 296.82 or ICD-10-CM codes: F30, F31), major depressive disorder (ICD-9-CM codes: 296.2, 296.3, 300.3, 311 or ICD-10-CM codes: F32, F33, F34), attention-deficit hyperactivity disorder (ADHD) (ICD-9-CM code: 314 or ICD-10-CM code: F90), autism spectrum disorder (ASD) (ICD-9-CM codes: 299.0, 299.8, 299.9 or ICD-10-CM codes: F84.0, F84.5, F84.8, F84.9), alcohol use disorder (AUD) (ICD-9-CM codes: 291, 303.0, 303.9, 305.0 or ICD-10-CM code: F10) and substance use disorder (SUD) (ICD-9-CM codes: 292, 304, 305 except 305.0 and 305.1 or ICD-10-CM codes: F11, F12, F13, F14, F15, F16, F18, F19), which are also associated with suicide risk (Catala-Lopez *et al.*, 2022; Cheng *et al.*, 2023; Hirvikoski *et al.*, 2016; Rizk *et al.*, 2021), these major comorbidities also were investigated during the follow-up period for further evaluation of the effect of comorbidities on the risk of suicide. These psychiatric disorders were diagnosed at least twice by board-certified psychiatrists. Finally, the history of psychiatric hospitalization owing to OCD based on the diagnostic code of OCD at discharge was assessed during the follow-up.

### Statistical analysis

For between-group comparisons in grouping data, repeated measures analysis of variance analyses with the general linear models were used for continuous variables, and conditional logistic regressions were used for nominal variables. Time-dependent Cox regression models with adjustment for sex, birth year, income, level of urbanization, psychiatric comorbidities and CCI were used to calculate HR and 95% CI of subsequent suicide between groups. We assessed the association between the history of psychiatric hospitalization owing to OCD based on the diagnostic code of OCD at discharge and the suicide risk using the Cox regression models with adjustment for demographic characteristics, psychiatric comorbidities and CCI. The history of psychiatric hospitalization owing to OCD during the follow-up was categorized to four subgroups: never, <1/year, 1–2/year and >2/year. The use of the average number of hospitalizations, rather than the total number of hospitalizations, may help to avoid the immortal time bias. Furthermore, we investigated the roles of psychiatric comorbidities, including schizophrenia, bipolar disorder, major depressive disorder, ASD, ADHD, AUD and SUD, on the suicide among individuals with OCD compared with the comparison group using the Cox regression models with adjustment for sex, birth year, income, level of urbanization and CCI. The proportional hazards assumptions were verified using the log-minus-log plots, resulting in no considerable violation. A two-tailed *p* value of <.05 was considered statistically significant. All data processing and statistical analyses were performed using the Statistical Analysis Software Version 9.1 (SAS Institute, Cary, NC, USA).

## Results

In all, 56,977 individuals with OCD and 227,908 age-/sex-matched non-OCD individuals were included in the current study (Table 1, Fig. 1). Of 56,977 individuals with OCD, 9654 (16.94%) were comorbid with schizophrenia, 8189 (14.37%) with bipolar disorder, 37,019 (64.97%) with major depressive disorder, 1768 (3.10%) with ASD, 3204 (5.62%) with ADHD, 2028 (3.56%) with AUD and 1622 (2.85%) with SUD (Table 1). In addition, 48,870 (85.77%) individuals with OCD were classified as the never hospitalization group, 5773 (10.13%) as the <1 time/year hospitalization group, 1295 (2.27%) as the 1–2 times/year hospitalization group and 1039 (1.82%) as the >2 times/year hospitalization group (Table 1).

**Table 1.** Demographic characteristics of patients with OCD and matched controls

	Patients with OCD *n* = 56,977	Comparison group *n* = 227,908	*p*-value
Birth year (*n*, %)			
–1950	1116 (1.96)	4464 (1.96)	
1951–1960	8507 (14.93)	34,028 (14.93)	
1961–1970	13,976 (24.53)	55,904 (24.53)	
1971–1980	12,879 (22.60)	51,516 (22.60)	
1981–1990	10,065 (17.67)	40,260 (17.67)	
1991–2000	6460 (11.34)	25,840 (11.34)	
2000–	3974 (6.97)	15,896 (6.97)	
Male (*n*, %)	28,727 (50.42)	114,908 (50.42)	
Monthly income (*n*, %)			<0.001
0–1000 USD	21,423 (37.60)	82,375 (36.14)	
1001–1800 USD	19,781 (34.72)	87,821 (38.53)	
≥1801 USD	15,773 (27.68)	57,712 (25.32)	
Level of urbanization (*n*, %)			<0.001
1 (Urban)	39,816 (69.88)	156,660 (68.74)	
2	12,062 (21.17)	49,372 (21.66)	
3	3509 (6.16)	14,728 (6.46)	
4 (Rural)	1590 (2.79)	7148 (3.14)	
Frequency of OCD-related psychiatric hospitalization (*n*, %)			
Never	48,870 (85.77)		
<1/year	5773 (10.13)		
1–2/year	1295 (2.27)		
>2/year	1039 (1.82)		
CCI (*n*, %)			<0.001
0	14,042 (24.65)	92,392 (40.54)	
1–2	25,040 (43.95)	91,939 (40.34)	
>2	17,895 (31.41)	43,577 (19.12)	
Psychiatric comorbidities (*n*, %)			
Schizophrenia	9654 (16.94)	1897 (0.83)	<0.001
Bipolar disorders	8189 (14.37)	1647 (0.72)	<0.001
Major depressive disorder	37,019 (64.97)	12,019 (5.27)	<0.001
ASD	1768 (3.10)	264 (0.12)	<0.001
ADHD	3204 (5.62)	1592 (0.70)	<0.001
AUD	2028 (3.56)	341 (0.60)	<0.001
SUD	1622 (2.85)	273 (0.48)	<0.001

OCD: obsessive compulsive disorder; USD: United State dollar; CCI: Charlson Comorbidity Index; ASD: autism spectrum disorder; ADHD: attention deficit hyperactivity disorder; AUD: alcohol use disorder; SUD: substance use disorder.

The Cox regression models with adjustment for demographic characteristics, psychiatric comorbidities and CCI showed that individuals with OCD (HR: 1.97, 95% CI: 1.57–2.48), including males (1.86, 1.37–2.53) and females (1.98, 1.38–2.84), were more likely to die by suicide during the follow-up compared with those without OCD (Table 2). We found a dose-dependent association between the frequency, including never (HR: 1.50, 95% CI: 1.24–1.81), <1 time/year (2.11, 1.59–2.79), 1–2 times/year (4.92, 3.50–6.93) and >2 times/year (8.06, 5.82–11.15), of psychiatric hospitalization owing to OCD and suicide risk among individuals with OCD compared with the comparison group (Table 3).

**Table 2.** Suicide risk between patients with OCD and matched controls

	All sample			Male sample			Female sample		
	Event (*n*)	Suicide rate (per 100,000 person-year)	HR# (95% CI)	Event (*n*)	Suicide rate (per 100,000 person-year)	HR# (95% CI)	Event (*n*)	Suicide rate (per 100,000 person-year)	HR# (95% CI)
Patients with OCD	595	70.9	**1.97 (1.57**–**2.48)**	306	72.5	**1.86 (1.37**–**2.53)**	289	69.3	**1.98 (1.38**–**2.84)**
Comparison group	392	11.6	1.00 (ref. group)	246	14.5	1.00 (ref. group)	146	8.7	1.00 (ref. group)

OCD: obsessive compulsive disorder; HR: hazard ratio; CI: confidence interval; CCI: Charlson Comorbidity Index.

# adjusting for sex, birth year, income, level of urbanization, psychiatric comorbidities and CCI.

Bold type indicates the statistical significance.

**Table 3.** Suicide risk between patients with OCD at different frequencies of psychiatric hospitalization and matched controls

	Suicide (HR, 95%)#
Comparison group	1.00 (ref.)
Patients with OCD	
Never	**1.50 (1.24–1.81)**
<1/year	**2.11 (1.59–2.79)**
1–2/year	**4.92 (3.50–6.93)**
>2/year	**8.06 (5.82–11.15)**
P for trend	<.001

OCD: obsessive compulsive disorder.

# adjusting for sex, birth year, income, level of urbanization, psychiatric comorbidities and CCI.

Bold type indicates the statistical significance

In addition, individuals with OCD who were comorbid with any major psychiatric disorder, including schizophrenia (HR: 3.40, 95% CI: 2.71–4.26), bipolar disorder (3.27, 2.57–4.15), major depressive disorder (6.47, 5.53–7.57), ASD (2.23, 1.19–4.18), AUD (2.68, 1.88–3.82) and SUD (3.12, 2.27–4.29), were associated with the further elevated risk of suicide compared with the comparison group (Table 4). Stratified by sex, we discovered the consistent findings between males and females on the associations between major psychiatric comorbidities, including schizophrenia (HR, 95% CI, male: 3.50, 2.61–4.68; female: 3.37, 2.34–4.83), bipolar disorder (2.71, 1.94–3.79; 3.79, 2.67–5.37), major depressive disorder (5.27, 4.27–6.52; 8.47, 6.66–10.78), AUD (2.16, 1.35–3.45; 4.12, 2.40–7.06) and SUD (2.64, 1.72–4.06; 3.88, 2.41–6.24), and the further suicide risk (Table 4). We additionally found that only males, but not females, with OCD comorbid with ASD (HR: 2.26, 95% CI: 1.12–4.58) and only females, but not males, with OCD comorbid with ADHD (2.41, 1.02–5.67) were more likely to die by suicide compared with the comparison group (Table 4).

**Table 4.** Suicide risk between patients with OCD with different psychiatric comorbidities and matched controls

	Suicide (HR, 95%)#		
	All sample	Male sample	Female sample
Comparison group	1.00 (ref.)	1.00 (ref.)	1.00 (ref.)
Patients with OCD			
Without any psychiatric comorbidity	**1.87 (1.52–2.31)**	**1.65 (1.24–2.19)**	**2.13 (1.56–2.91)**
With any psychiatric comorbidity	**2.43 (1.72–3.44)**	**2.49 (1.66–3.76)**	**2.36 (1.23–4.54)**
Comparison group	1.00 (ref.)	1.00 (ref.)	1.00 (ref.)
Patients with OCD			
Without schizophrenia	**1.97 (1.62–2.40)**	**1.82 (1.40–2.37)**	**2.15 (1.60–2.89)**
With schizophrenia	**3.40 (2.71–4.26)**	**3.50 (2.61–4.68)**	**3.37 (2.34–4.83)**
Comparison group	1.00 (ref.)	1.00 (ref.)	1.00 (ref.)
Patients with OCD			
Without bipolar disorder	**1.93 (1.59–2.34)**	**1.83 (1.41–2.36)**	**2.05 (1.53–2.76)**
With bipolar disorder	**3.27 (2.57–4.15)**	**2.71 (1.94–3.79)**	**3.79 (2.67–5.37)**
Comparison group	1.00 (ref.)	1.00 (ref.)	1.00 (ref.)
Patients with OCD			
Without major depressive disorder	**2.51 (1.98–3.18)**	**2.39 (1.79–3.20)**	**2.63 (1.74–3.98)**
With major depressive disorder	**6.47 (5.53–7.57)**	**5.27 (4.27–6.52)**	**8.47 (6.66–10.78)**
Comparison group	1.00 (ref.)	1.00 (ref.)	1.00 (ref.)
Patients with OCD			
Without ASD	**2.00 (1.65–2.41)**	**1.86 (1.45–2.40)**	**2.17 (1.63–2.89)**
With ASD	**2.23 (1.19–4.18)**	**2.26 (1.12–4.58)**	2.08 (0.50**–**8.68)
Comparison group	1.00 (ref.)	1.00 (ref.)	1.00 (ref.)
Patients with OCD			
Without ADHD	**2.00 (1.66–2.42)**	**1.87 (1.45–2.40)**	**2.17 (1.62–2.89)**
With ADHD	1.56 (0.92**–**2.66)	1.31 (0.67**–**2.58)	**2.41 (1.02–5.67)**
Comparison group	1.00 (ref.)	1.00 (ref.)	1.00 (ref.)
Patients with OCD			
Without AUD	**1.95 (1.61–2.36)**	**1.82 (1.41–2.35)**	**2.11 (1.58–2.82)**
With AUD	**2.68 (1.88–3.82)**	**2.16 (1.35–3.45)**	**4.12 (2.40–7.06)**
Comparison group	1.00 (ref.)	1.00 (ref.)	1.00 (ref.)
Patients with OCD			
Without SUD	**1.94 (1.60–2.34)**	**1.80 (1.39–2.32)**	**2.11 (1.58–2.83)**
With SUD	**3.12 (2.27–4.29)**	**2.64 (1.72–4.06)**	**3.88 (2.41–6.24)**

OCD: obsessive compulsive disorder; ASD: autism spectrum disorder; ADHD: attention deficit hyperactivity disorder; AUD: alcohol use disorder; SUD: substance use disorder.

# Separate Cox regression models with adjustment of sex, birth year, income, level of urbanization and CCI.

Bold type indicates the statistical significance

## Discussion

The study findings supported our initial hypothesis that individuals with OCD were at higher risk of death by suicide during the follow-up period compared with the non-OCD individuals, independent of the existence of major psychiatric comorbidities. Additionally, individuals with more frequent OCD-related psychiatric hospitalization, potentially indicative of the severity of their OCD, were at greater risk of suicide.

As mentioned in the introduction, Fernandez de la Cruz *et al.* (2017) reported that OCD was associated with suicide death, even after adjustment for major affective disorders or psychotic disorders (OR: 7.51, 95% CI: 6.58–8.71; 8.81, 7.76–10.00, respectively). They also discovered that within their OCD group, 179 of the 545 individuals (32.84%) who died by suicide had a documented history of a previous suicide attempt (Fernandez de la Cruz *et al.*, 2017). Surprisingly, their study revealed that although major affective disorders and psychotic disorders increased the risk of a subsequent suicide attempt (HRs, 95% CI: 2.03, 1.88–2.20; 1.46, 1.35–1.58, respectively), they did not significantly influence the risk of death by suicide (0.94, 0.75–1.18; 1.05, 0.82–1.35, respectively) (Fernandez de la Cruz *et al.*, 2017). Using data from Danish registers, Meier *et al.*, (2016) similarly indicated that the suicide-specific mortality rate ratio (MRR) in patients with OCD was 3.02 (95% CI: 1.85–4.63). They further revealed that individuals with OCD and comorbid depression had a higher MRR of 2.47 (95% CI: 1.57–3.65) than did individuals with OCD alone (1.88, 1.27–2.67) (Meier *et al.*, 2016). The results of Fernandez de la Cruz *et al.* (2017) and Meier *et al.* (2016) were partially compatible with our finding that individuals with OCD had higher risk of suicide compared with the comparison group independent of major psychiatric comorbidities, including schizophrenia, bipolar disorder, major depressive disorder, ASD, ADHD, AUD and SUD. Additionally, our findings indicated that OCD severity, as indicated by the frequency of psychiatric hospitalizations due to OCD, was positively associated with suicide risk. Furthermore, the psychiatric comorbidity that resulted in the highest suicide risk was identified to be major depressive disorder, followed by schizophrenia, bipolar disorder, SUD, AUD and ASD, in individuals with OCD.

We propose several pathomechanisms to elucidate the association between OCD and suicide. First, the core symptoms of OCD, specifically obsessions and compulsions, are associated with suicide-related symptoms, including suicidal thoughts, suicide attempts and suicide death (Angelakis *et al.*, 2015; Kim *et al.*, 2023; Velloso *et al.*, 2016). A network analysis conducted on a cohort of 444 patients with OCD revealed that suicidal thoughts were directly associated with distress related to compulsive behaviours, the amount of time spent engaging in compulsive behaviours and distress stemming from unacceptable obsessive thoughts (Kim *et al.*, 2023). Angelakis *et al.* (2015) reported that the severity of obsessions was positively correlated with an elevated level of suicidality, including suicidal thoughts and suicide attempts, in individuals with OCD. Velloso *et al.* (2016) emphasized that the severity of aggression, sexual or religious obsession, and symmetry or ordering compulsion was particularly associated with suicide risk. Our findings also support OCD as an independent risk factor for death by suicide, even when comorbid psychiatric disorders have been adjusted for. Second, as specified in the introduction, psychiatric comorbidities, such as schizophrenia and major depressive disorder, play a pivotal role in the association between OCD and suicide (Angelakis *et al.*, 2015; Meier *et al.*, 2016; Ouazzani Housni Touhami *et al.*, 2023). Our study revealed that individuals with OCD who had comorbid bipolar disorder had the highest HR for death by suicide (12.13). Furthermore, a genome-wide association study with 383 people who had bipolar disorder calculated the polygenic risk score (PRS) for schizophrenia, bipolar disorder, major depressive disorder and OCD, but surprisingly revealed that only the PRS for OCD was significantly associated with lifetime suicide attempts (Lee *et al.*, 2022). Lee *et al.* (2022) further reported that the OCD-PRS of individuals who had made multiple attempts was higher than that of those with a history of a single suicide attempt. Di Salvo *et al.* (2020) demonstrated that individuals with comorbid OCD and bipolar disorder were more prone to attempting suicide by using violent methods compared with those with OCD only. Finally, accumulating evidence indicates a common genetic background between OCD and suicide (Krebs *et al.*, 2021). The Swedish Child and Adolescent Twin Longitudinal Study revealed that genetic factors accounted for 74.6% and nonshared environmental factors accounted for 25.4% of the association between OCD and suicide attempts (Krebs *et al.*, 2021). Sidorchuk *et al.* (2021) reported that the familial coaggregation of OCD and death by suicide was primarily attributed to additive genetic factors (65.8%) and nonshared environmental factors (34.2%), with a negligible contribution from shared environmental factors.

Several limitations of the present study should be acknowledged and addressed. First, the low prevalence of OCD in Taiwan has been reported both in the field study (0.4/100) and in a national population-based study (1-year prevalence: 65.05/10^5^) (Huang *et al.*, 2014; Weissman *et al.*, 1994). Our study may have underestimated the true prevalence of OCD. This is because we relied on data from individuals who sought medical consultation and treatment, meaning that individuals with OCD who did not seek medical help were not included in the database from which we acquired data. Studies have reported that OCD is often underdiagnosed and undertreated (Fontenelle and Yucel, 2019; Hirschtritt *et al.*, 2017). Individuals with OCD who were included in our study may be more severely affected since they have appeared in the medical system. Whether our findings may be generalized to the general population, which includes those with mild and subclinical OCD, or other ethnicities would need further investigation. Second, the common comorbidities with schizophrenia, major affective disorders, AUD, SUD, ADHD and ASD with OCD may reflect the heterogeneity of psychopathology in the patients with OCD in the present study (Buckley *et al.*, 2009; Eisen and Rasmussen, 1993; Ruscio *et al.*, 2010). However, adjusting for the presence of psychiatric comorbidities, we still found OCD to be an independent risk factor for subsequent suicide. Further clinical studies may be required to delicately elucidate the separate contribution of the psychopathology of OCD per se or OCD-related comorbidities to suicide. Third, the database used in this study lacked information on environmental factors, psychosocial stress, lifestyle and other potential variables that could influence the study’s outcomes. Therefore, we could not comprehensively investigate the contributions of these factors to suicide risk.

In conclusion, our study provides compelling evidence that individuals with OCD had higher risk of suicide during the follow-up period compared with those without OCD. Furthermore, OCD emerged as an independent risk factor for suicide, regardless of the existence of major psychiatric disorders, including schizophrenia, bipolar disorder, major depressive disorder, ASD and ADHD. Furthermore, the severity of OCD, as indicated by the frequency of psychiatric hospitalizations, was associated with increased suicide risk. Although our findings shed light on the complex OCD–suicide relationship, the exact mechanisms warrant further investigation. A suicide prevention program specific to individuals with OCD may be developed in clinical practice in the future.

## Data Availability

The NHIRD was released and audited by the Department of Health and Bureau of the NHI Program for the purpose of scientific research (https://www.apre.mohw.gov.tw/). The NHIRD can be accessed through a formal application that is regulated by the Health and Welfare Data Science Center of Ministry of Health and Welfare, Taiwan.
